# DOA Estimation under Unknown Mutual Coupling and Multipath with Improved Effective Array Aperture

**DOI:** 10.3390/s151229832

**Published:** 2015-12-08

**Authors:** Yuexian Wang, Matthew Trinkle, Brian W.-H. Ng

**Affiliations:** University of Adelaide Radar Research Centre, School of Electrical and Electronic Engineering, The University of Adelaide, Adelaide, SA 5005, Australia; mtrinkle@eleceng.adelaide.edu.au (M.T.); brian.ng@adelaide.edu.au (B.W.-H.N.)

**Keywords:** DOA estimation, coherent signals, mutual coupling, matrix reconstruction

## Abstract

Subspace-based high-resolution direction of arrival (DOA) estimation significantly deteriorates under array manifold perturbation and rank deficiency of the covariance matrix due to mutual coupling and multipath propagation, respectively. In this correspondence, the unknown mutual coupling can be circumvented by the proposed method without any passive or active calibration process, and the DOA of the coherent signals can be accurately estimated accordingly. With a newly constructed matrix, the deficient rank can be restored, and the effective array aperture can be extended compared with conventional spatial smoothing. The proposed method achieves a good robustness and DOA estimation accuracy with unknown mutual coupling. The simulation results demonstrate the validity and efficiency of the proposed method.

## 1. Introduction

In array signal processing, direction of arrival (DOA) estimation of incident signals is an important research issue. Many high-resolution methods based on the orthogonality of subspaces have been studied extensively over the years, such as MUSIC [1] and ESPRIT [2]. Most of these algorithms assume that the array manifold is known and the signals are uncorrelated. However, these two prerequisites are not always guaranteed in practice as mutual coupling between the elements is an intrinsic characteristic of antenna arrays and multipath propagation caused by reflective surfaces are very common in urban areas, both resulting in a failure of subspace-based methods [3,4].

In electromagnetic engineering, the mutual coupling matrix can be calculated using techniques such as finite element or method of moment techniques [5,6,7]. However, these calculation depends heavily on the physical shape of the antennas and are specific for each antenna array. It is thus often preferable to carry out a joint estimation of the mutual coupling matrix and signal DOA from a signal processing perspective. Weiss and Friedlander [3] proposed an iterative algorithm that compensates for mutual coupling and gain and phase perturbations, in uniform linear and circular arrays. Subsequently, Sellone *et al.* proposed another iterative method, which alternatingly minimises a cost function with respect to two complex valued symmetric Toeplitz matrices and a complex Hermitian Toeplitz matrix [8]. Compared with the method proposed in [3], this method is less sensitive to the array perturbations and can get an effective DOA estimate without a preliminary estimate in many cases. However, the computational complexities of both are high because the non-linear multidimensional search process is random in nature.

More recently, several DOA estimation algorithms which are not sensitive to mutual coupling have been developed [9,10,11]. By setting a group of sensors as auxiliary sensors, the DOAs can be directly estimated without compensating for mutual coupling. Moreover, the result can be refined iteratively by estimating the mutual coupling coefficients for compensation. The main advantage of these methods is that no iteration is required but the aperture is reduced by making some sensors auxiliary.

All the algorithms reviewed above are only effective in a scenario where the incident signals are uncorrelated to each other and do not undergo multipath propagation. In a typical wireless communication system where multipath propagation is unavoidable, these algorithms may not be effective.

To tackle these two thorny issues, a class of auto-calibration methods, which are able to estimate the mutual coupling coefficients and DOAs of coherent signals jointly, has been addressed in [12,13]. In [12], the mutual coupling coefficients and coherent DOAs can be estimated jointly in an alternating manner, but this results in a large computational complexity for the nonlinear multidimensional search and is not applicable in some systems due to the requirement of pilot symbols. A noniterative optimisation method is given in [13] to estimate the DOAs of mixed signals which include coherent signals, but the mutual coupling coefficients have to be estimated from uncorrelated signals first. Hence, it may not be effective when there are only coherent signals. Recently, a spatial smoothing scheme studied in [14] estimates the DOAs of coherent signals and is insensitive to unknown mutual coupling, but only the forward smoothing was considered and the conjugate information contained within the signals was not utilised. More recently, Toeplitz matrix reconstruction directly in data domain [15] showed good performance for real-time applications in decorrelating coherent signals without mutual coupling compensation, but at the cost of halving the effective array aperture which is very limiting in practice.

In this correspondence, we propose a new matrix reconstruction method which is insensitive to mutual coupling and has an improved aperture after rank restoration. The main differences between our method and the methods in [14,15] lie in:

1. Our method exploits the conjugate of the received data for the purpose of rank restoration, which gives rise to more degrees of freedom (DOF) as well as an extended aperture, while both the algorithms in [14,15] ignore such information, which significantly restricts the identifiability of DOA estimates.
2. Our method makes use of all entries of the covariance matrix of the middle subarray, and the performance in terms of estimation resolution will thus be improved, whereas the spatial smoothing technique adopted in [14], only averages the covariance matrices of the subarrays but does not take advantage of the cross correlations between them, and thus the estimation performance will be compromised to some extent.
3. Our approach offers a clear advantage in how it adapts to changes in the number of signals, whereas the method in [15] has a fixed number of DOF regardless of the number of signals.

These differences combine together so that our method can offer better estimation performance than existing techniques that incorporate forward-only averaging.

The rest of the paper is organised as follows. In Section 2, the signal model when coherent signals and mutual coupling coexist is presented. In Section 3, we develop a preprocessing strategy to estimate the DOAs of the coherent signals which is blind to the mutual coupling effects and does not require array calibration. In Section 4, simulation results show the validity and efficiency of our proposed method. Finally, some concluding remarks are given in Section 5.

Throughout this paper, the notations that will be used are listed as follows. The operators ${\left(\cdot \right)}^{T}$, ${\left(\cdot \right)}^{*}$, ${\left(\cdot \right)}^{H}$, ${\left(\cdot \right)}^{-1}$, E[·], ${∥\cdot ∥}_{F}$, ⌈·⌉, and ⌊·⌋ denote the operation of transpose, conjugate, conjugate transpose, inverse, expectation, the Frobenius norm, ceiling, and flooring of a decimal number, respectively. The symbol $\text{diag}\{z_{1},z_{2}\}$ represents a diagonal matrix with diagonal entries $z_{1},z_{2}$ and $\text{blkdiag}\{Z_{1},Z_{2}\}$ represents a block diagonal matrix with diagonal entries $Z_{1},Z_{2}$. The symbol rank(Z) denotes the rank of a matrix **Z**. The symbol Z(a:b,c:d) denotes a constructed submatrix by the entries from rows *a* to *b* and columns *c* to *d* of Z, and the symbol Z(a,b) denotes the entry in the *a*-th row and *b*-th column of **Z**.

## 2. Problem Formulation

Consider a number of *N* narrowband far-field coherent signals impinging on a uniform linear array (ULA) with *M* identical omnidirectional sensors. Assume that these signals are classified into *K* groups, which result from *K* statistically uncorrelated far-field sources $s_{k}\left(t\right),k=1,2,\cdots ,K$ with $P_{k}$ multipath signals for each source. In the *k*-th coherent group, the signal coming from direction $\theta_{kp}$, $p=1,2,\cdots ,P_{k}$ corresponds to the *p*-th multipath propagation of the source $s_{k}\left(t\right)$, and the complex fading coefficient is $\alpha_{kp}$. It is readily seen that the total number of coherent signals satisfies $N=\sum_{k=1}^{K}P_{k}$. Considering the effect of mutual coupling between the array elements, the corresponding M×1 array output vector is then given by

(1) $$\begin{array}{cc} x(t) & =\sum_{k=1}^{K}\sum_{p=1}^{P_{k}}Ca\left(\theta_{kp}\right)\alpha_{kp}s_{k}\left(t\right)+n\left(t\right)\\ & =CA\Gamma s(t)+n(t) \end{array}$$

where $a\left(\theta \right)={\left[\begin{array}{c} 1,e^{j\frac{2\pi d}{\lambda }\sin\;\theta },\cdots ,e^{j\frac{2\pi (M-1)d}{\lambda }\sin\;\theta } \end{array}\right]}^{T}\in C^{M}$ is the steering vector with *λ* and *d* being the wavelength of carrier signal and the spacing between adjacent elements, respectively, **C** denotes the mutual coupling matrix (MCM), $A=\left[\begin{array}{c} A_{1},\cdots ,A_{K} \end{array}\right]$ with $A_{k}=\left[\begin{array}{c} a\left(\theta_{k1}\right),\cdots ,a\left(\theta_{kP_{k}}\right) \end{array}\right]$, $\text{Γ}\;=\;\text{blkdiag}\{\alpha_{1},\cdots ,\alpha_{K}\}$ with $\alpha_{k}={\left[\begin{array}{c} \alpha_{k1},\cdots ,\alpha_{kP_{k}} \end{array}\right]}^{T}$ containing attenuation information of the *k*-th coherent group, $s\left(t\right)={\left[\begin{array}{c} s_{1}\left(t\right),\cdots ,s_{K}\left(t\right) \end{array}\right]}^{T}$, and n(t) is white Gaussian noise with the power $\sigma_{n}^{2}$ for each entry. Besides, we assume that the array manifold **A** is unambiguous, *i.e.*, the steering vectors ${\left\{a\left(\theta_{i}\right)\right\}}_{i=1}^{N}$ are linearly independent for any set of distinct ${\left\{\theta_{i}\right\}}_{i=1}^{N}$.

**Figure 1:**
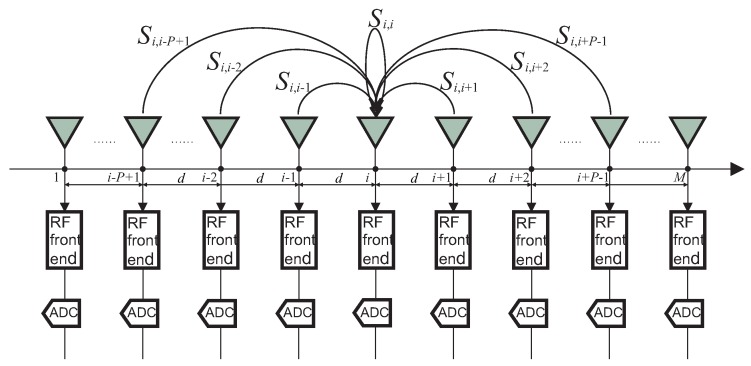
Mutual coupling to the *i*-th element of a uniform linear array.

As described in [3,12,13,14,15], it is often sufficient to consider the ULA coupling model that has just finite non-zero coefficients, and a banded symmetric Toeplitz matrix can be used to model mutual coupling. To elaborate on the structure of the MCM, we consider the coupling effects to the *i*-th element shown in Figure 1 as a instance, where $S_{i,u}=c_{\left|u-i\right|}A\left(u,:\right)\Gamma s\left(t\right)$, 1≤i,u≤M, is the coupling contribution from the *u*-th sensor to the *i*-th sensor with $c_{\left|u-i\right|}$ being the mutual coupling coefficient. It is known that the effect of mutual coupling between two sensor elements is inversely related to their distance and can be ignored when the separation is more than a few wavelengths. More precisely, we assume that when the distance between two sensors is more than Pd where *d* is the inter-element spacings in a uniform linear array, the mutual coupling coefficient can be approximated as zero. We also assume that the mutual coupling coefficients only depend on the distance between two sensor elements. Based on these assumptions, the observed output at the *i*-th sensor can be expressed as

(2) xi(t)=∑u=i−P+11≤i,u≤Mi+P−1Si,u+ni(t)=01×(i−P),cP−1,⋯,c1,1,c1,⋯,cP−1,01×(M−i−P+1)AΓs(t)+ni(t)

where $n_{i}\left(t\right)$ is the additive noise at the *i*-th sensor. Considering mutual coupling for all the *M* sensors and stacking $x_{1}\left(t\right),x_{2}\left(t\right),\cdots ,x_{M}\left(t\right)$ in a column, one obtains Equation (1) where

(3) $$\begin{array}{cc} C & =\left[\begin{array}{cccccc} 1 & c_{1} & \cdots & c_{P-1} & & 0\\ c_{1} & 1 & c_{1} & \cdots & \ddots & \\ \vdots & c_{1} & 1 & c_{1} & \cdots & c_{P-1}\\ c_{P-1} & \cdots & \ddots & \ddots & \ddots & \vdots \\ & \ddots & \cdots & c_{1} & 1 & c_{1}\\ 0 & & c_{P-1} & \cdots & c_{1} & 1 \end{array}\right]\\ & =\text{Toeplitz}\left\{\left[1,c_{1},\cdots ,c_{P-1},0_{1\times (M-P)}\right]\right\} \end{array}$$

with $0<|c_{1}\left|,\right|c_{2}\left|,\cdots ,\right|c_{P-1}|<c_{0}=1$.

From Equation (1), the array covariance matrix is given by

(4) $$\begin{array}{cc} R_{x} & =E\left[x\left(t\right)x^{H}\left(t\right)\right]=CA\Gamma R_{s}\Gamma^{H}A^{H}C^{H}+\sigma_{n}^{2}I_{M} \end{array}$$

where $R_{s}=E\left[s\left(t\right)s^{H}\left(t\right)\right]=\mathrm{diag}\left\{\sigma_{1}^{2},\cdots ,\sigma_{K}^{2}\right\}$ is the signal covariance matrix, and $I_{M}$ represents an M×M identity matrix. In the case of finite snapshots, the array covariance matrix can be calculated as ${\hat{R}}_{x}=\frac{1}{L}\sum_{t=1}^{L}x\left(t\right)x^{H}\left(t\right)$, where *L* is the total number of snapshots.

## 3. DOA Estimation without Mutual Coupling Calibration

### 3.1. Mutual Coupling Circumvention

**Figure 2:**
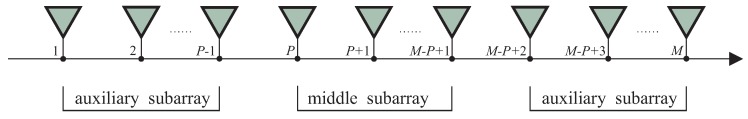
Allocation of the middle subarray and auxiliary subarray.

Referring to [9,10,11], we know that the middle subarray, defined as the middle M−2P+2 elements, is insensitive to mutual coupling, and its actual steering vector is equivalent to an ideal one (*i.e.*, no coupling effect) scaled by a scalar. In order to combat signal coherency as well as effect of unknown mutual coupling in the ULA, we only make use of the output of the middle subarray by virtue of their steering vector. All other elements are defined to belong to auxiliary subarrays as shown in Figure 2. Inspired by [9], a selection matrix **J** is defined as

(5) $$\begin{array}{cc} J & =\left[\begin{array}{c} 0_{\left(M-2P+2)\times (P-1\right)},I_{M-2P+2},0_{\left(M-2P+2)\times (P-1\right)} \end{array}\right] \end{array}$$

and the covariance matrix of the the middle subarray is then given by

(6) $$\begin{array}{cc} {\tilde{R}}_{x} & =JR_{x}J^{H}=JCA\Gamma R_{s}\Gamma^{H}A^{H}C^{H}J^{H}+\sigma_{n}^{2}JJ^{H}\\ & =\tilde{C}A\Gamma R_{s}\Gamma^{H}A^{H}{\tilde{C}}^{H}+\sigma_{n}^{2}I_{M-2P+2} \end{array}$$

where

(7) $$\begin{array}{cc} \tilde{C} & =\left[\begin{array}{cccccccc} c_{P-1} & \cdots & 1 & \cdots & c_{P-1} & 0 & \cdots & 0\\ 0 & c_{P-1} & \cdots & 1 & \cdots & c_{P-1} & \cdots & 0\\ \vdots & \ddots & \ddots & \cdots & \ddots & \cdots & \ddots & \vdots \\ 0 & \cdots & 0 & c_{P-1} & \cdots & 1 & \cdots & c_{P-1} \end{array}\right]. \end{array}$$

According to the selected MCM and signal models above, one has the following parameterisation for joint estimation of MCM and DOAs:

(8) $$\begin{array}{cc} \tilde{C}a\left(\theta \right) & =\left[\begin{array}{c} c_{P-1}+\cdots +\beta^{P-1}+\cdots +c_{P-1}\beta^{2P-2}\\ c_{P-1}\beta +\cdots +\beta^{P}+\cdots +c_{P-1}\beta^{2P-1}\\ \vdots \\ c_{P-1}\beta^{M-2P+1}+\cdots +\beta^{M-P}+\cdots +c_{P-1}\beta^{M-1} \end{array}\right]\\ & =\left(c_{P-1}+\cdots +\beta^{P-1}+\cdots +c_{P-1}\beta^{2P-2}\right)\left[\begin{array}{c} 1\\ \beta \\ \vdots \\ \beta^{M-2P+1} \end{array}\right]\\ & =\mu \left(\theta \right)\tilde{a}\left(\theta \right) \end{array}$$

with $\mu \left(\theta \right)=c_{P-1}+\cdots +\beta^{P-1}+\cdots +c_{P-1}\beta^{2P-2}$, $\beta =e^{j\frac{2\pi d}{\lambda }\sin\;\theta }$, and $\tilde{a}\left(\theta \right)={\left[1,\beta ,\cdots ,\beta^{M-2P+1}\right]}^{T}$. It should be noted that generally μ(θ) in Equation (8) is assumed to be nonzero. Hence, the covariance matrix of the middle array in Equation (6) can be rewritten as

(9) $$\begin{array}{cc} {\tilde{R}}_{x} & =\left[\begin{array}{c} \tilde{a}\left(\theta_{1}\right),\tilde{a}\left(\theta_{2}\right),\cdots ,\tilde{a}\left(\theta_{N}\right) \end{array}\right]\mathrm{diag}\left\{\mu \left(\theta_{1}\right),\mu \left(\theta_{2}\right),\cdots ,\mu \left(\theta_{N}\right)\right\}\Gamma R_{s}\\ & \;\times \Gamma^{H}\mathrm{diag}{\left\{\mu \left(\theta_{1}\right),\mu \left(\theta_{2}\right),\cdots ,\mu \left(\theta_{N}\right)\right\}}^{H}{\left[\begin{array}{c} \tilde{a}\left(\theta_{1}\right),\tilde{a}\left(\theta_{2}\right),\cdots ,\tilde{a}\left(\theta_{N}\right) \end{array}\right]}^{H}+\sigma_{n}^{2}I_{M-2P+2}\\ & =\tilde{A}D\Gamma R_{s}\Gamma^{H}D^{H}{\tilde{A}}^{H}+\sigma_{n}^{2}I_{M-2P+2}\\ & =\tilde{A}\tilde{\Gamma }R_{s}{\tilde{\Gamma }}^{H}{\tilde{A}}^{H}+\sigma_{n}^{2}I_{M-2P+2} \end{array}$$

where $\tilde{A}=\left[\begin{array}{c} \tilde{a}\left(\theta_{1}\right),\tilde{a}\left(\theta_{2}\right),\cdots ,\tilde{a}\left(\theta_{N}\right) \end{array}\right]$, $D=\mathrm{diag}\{\mu \left(\theta_{1}\right),\mu \left(\theta_{2}\right),\cdots ,\mu \left(\theta_{N}\right)\}$, and $\tilde{\Gamma }=D\Gamma$ is still a N×K block diagonal matrix. This indicates that the middle subarray, which involves M−2P+2 sensors, is insensitive to mutual coupling but is still affected by signal coherency.

### 3.2. DOA Estimation of Coherent Signals

In this section, we will propose a rank restoration algorithm using an improved matrix reconstruction technique for DOA estimation of coherent signals in the presence of unknown mutual coupling.

Here a new matrix from the *i*-th row of ${\tilde{R}}_{x}$ is constructed as

(10) $$\begin{array}{cc} & \;\;B(i)\\ & ≜\left[\begin{array}{cccccccc} {\tilde{R}}_{x}\left(i,m\right) & {\tilde{R}}_{x}\left(i,m+1\right) & \cdots & {\tilde{R}}_{x}\left(i,m+q-1\right) & {\tilde{R}}_{x}^{*}\left(i,1\right) & {\tilde{R}}_{x}^{*}\left(i,2\right) & \cdots & {\tilde{R}}_{x}^{*}\left(i,q\right)\\ {\tilde{R}}_{x}\left(i,m-1\right) & {\tilde{R}}_{x}\left(i,m\right) & \cdots & {\tilde{R}}_{x}\left(i,m+q-2\right) & {\tilde{R}}_{x}^{*}\left(i,2\right) & {\tilde{R}}_{x}^{*}\left(i,3\right) & \cdots & {\tilde{R}}_{x}^{*}\left(i,q+1\right)\\ \vdots & \vdots & \ddots & \vdots & \vdots & \vdots & \ddots & \vdots \\ {\tilde{R}}_{x}\left(i,1\right) & {\tilde{R}}_{x}\left(i,2\right) & \cdots & {\tilde{R}}_{x}\left(i,q\right) & {\tilde{R}}_{x}^{*}\left(i,m\right) & {\tilde{R}}_{x}^{*}\left(i,m+1\right) & \cdots & {\tilde{R}}_{x}^{*}\left(i,m+q-1\right) \end{array}\right] \end{array}$$

where q=M−2P+3−m. From Equations (1) and (9), the received signal ${\tilde{x}}_{i}\left(t\right)$ at the *i*-th element of the middle array is

(11) $$\begin{array}{cc} {\tilde{x}}_{i}\left(t\right) & =\sum_{k=1}^{K}\sum_{p=1}^{P_{k}}\mu \left(\theta_{kp}\right)\alpha_{kp}\beta_{kp}^{i-1}s_{k}\left(t\right)+n_{i}\left(t\right),\;i=1,2,\cdots ,M-2P+2 \end{array}$$

where $\beta_{kp}=e^{j\frac{2\pi d}{\lambda }\sin\;\theta_{kp}}$ and $n_{i}\left(t\right)$ are the white Gaussian noise at the *i*-th sensor.

Then, the (i,j)-th entry of ${\tilde{R}}_{x}$ can be expressed as

(12) $$\begin{array}{cc} & \;\;{\tilde{R}}_{x}\left(i,j\right)\\ & =E\left[{\tilde{x}}_{i}\left(t\right){\tilde{x}}_{j}^{*}\left(t\right)\right]\\ & =E[\left(\sum_{k=1}^{K}\sum_{p=1}^{P_{k}}\mu \left(\theta_{kp}\right)\alpha_{kp}\beta_{kp}^{i-1}s_{k}\left(t\right)+n_{i}\left(t\right)\right)\\ & \;\times \left(\sum_{k=1}^{K}\sum_{p=1}^{P_{k}}\mu^{*}\left(\theta_{kp}\right)\alpha_{kp}^{*}\beta_{kp}^{-(j-1)}s_{k}^{*}\left(t\right)+n_{j}^{*}\left(t\right)\right)]\\ & =\sum_{k=1}^{K}\sum_{l=1}^{P_{k}}\beta_{kl}^{-(j-1)}\mu^{*}\left(\theta_{kl}\right)\alpha_{kl}^{*}\sigma_{k}^{2}\sum_{p=1}^{P_{k}}\mu \left(\theta_{kp}\right)\alpha_{kp}\beta_{kp}^{i-1}+\sigma_{n}^{2}\delta_{i,j} \end{array}$$

where $\delta_{i,j}$ is the Kronecker delta. Substituting Equation (12) into the matrix in Equation (10) allows B(i) to be written as

(13) $$\begin{array}{cc} B(i) & =\left[\begin{array}{c} A_{c}\Phi^{1-m}b\left(i\right),A_{c}\Phi^{-m}b\left(i\right),\cdots ,A_{c}\Phi^{2-m-q}b\left(i\right),A_{c}b\left(i\right),A_{c}\Phi b\left(i\right),\cdots ,A_{c}\Phi^{q-1}b\left(i\right) \end{array}\right]\\ & \;+\left[\begin{array}{c} K_{i},L_{i} \end{array}\right]\\ & =A_{c}\left[\begin{array}{c} \Phi^{1-m}b\left(i\right),\Phi^{-m}b\left(i\right),\cdots ,\Phi^{2-m-q}b\left(i\right),b\left(i\right),\Phi b\left(i\right),\cdots ,\Phi^{q-1}b\left(i\right) \end{array}\right]+\left[\begin{array}{c} K_{i},L_{i} \end{array}\right]\\ & =A_{c}G\left(i\right)+\left[\begin{array}{c} K_{i},L_{i} \end{array}\right] \end{array}$$

where $A_{c}=F\left[\begin{array}{c} \tilde{a}\left(\theta_{1}\right),\tilde{a}\left(\theta_{2}\right),\cdots ,\tilde{a}\left(\theta_{N}\right) \end{array}\right]$, $F=\left[\begin{array}{c} I_{M-2P+3-q},0_{\left(M-2P+3-q)\times (q-1\right)} \end{array}\right]$, $b\left(i\right)={\left[b_{11}\left(i\right),\cdots ,b_{1P_{1}}\left(i\right),\cdots ,b_{K1}\left(i\right),\cdots ,b_{KP_{K}}\left(i\right)\right]}^{T}$, $b_{kp}\left(i\right)=\sigma_{k}^{2}\mu^{*}\left(\theta_{kp}\right)\alpha_{kp}^{*}\sum_{l=1}^{P_{k}}\mu \left(\theta_{kl}\right)\alpha_{kp}\beta_{kl}^{i-1}$, $G\left(i\right)=\left[\Phi^{1-m}b\left(i\right),\Phi^{-m}b\left(i\right),\cdots ,\Phi^{2-m-q}b\left(i\right),b\left(i\right),\Phi b\left(i\right),\cdots ,\Phi^{q-1}b\left(i\right)\right]$ with $\Phi =\text{diag}\left\{\beta_{11},\cdots ,\beta_{1P_{1}},\cdots ,\beta_{K1},\cdots ,\beta_{KP_{K}}\right\}$, $K_{i},L_{i}\in R^{m\times q}$, and their entries are defined as $K_{i}\left(u,v\right)=\left\{\begin{array}{cc} \sigma_{n}^{2},\; & u-v+i=m\\ 0,\; & \text{otherwise} \end{array}\right.$, $L_{i}\left(u,v\right)=\left\{\begin{array}{cc} \sigma_{n}^{2},\; & u+v=i+1\\ 0,\; & \text{otherwise} \end{array}\right.$, respectively.

It is known that the effect of having only a finite number of snapshots, especially when the number is small, may cause biased estimates of subspaces. To further restore the rank and make the matrix reconstruction method more robust against the effects of noise and finite snapshots, all the M−2P+2 rows of ${\tilde{R}}_{x}$ are then exploited to construct the following square matrix

(14) $$\begin{array}{cc} \bar{R} & =\sum_{i=1}^{M-2P+2}B\left(i\right)B^{H}\left(i\right)\\ & =A_{c}\left(\sum_{i=1}^{M-2P+2}G\left(i\right)G^{H}\left(i\right)\right)A_{c}^{H}+\sum_{i=1}^{M-2P+2}\left(K_{i}K_{i}^{H}+L_{i}L_{i}^{H}\right) \end{array}$$

It can be readily verified that $\sum_{i=1}^{M-2P+2}\left(K_{i}K_{i}^{H}+L_{i}L_{i}^{H}\right)=\eta I_{m}$ where

(15) $$\eta =\left\{\begin{array}{cc} 2\left(m-1\right)\sigma_{n}^{2},\; & m>q\\ 2q\sigma_{n}^{2},\; & m\leq q \end{array}\right.$$

Defining $R_{d}≜A_{c}\left(\sum_{i=1}^{M-2P+2}G\left(i\right)G^{H}\left(i\right)\right)A_{c}^{H}$, then $\bar{R}=R_{d}+\eta I_{m}$.

Next we examine whether the rank of $R_{d}$ has been restored sufficiently to resolve all *N* incident coherent signals.

**Proposition 1.**  *When* m≥N+1 *and* $2q\geq P_{max}$, $rank(R_{d})=N$ *for K groups of coherent signals, where* $P_{max}=max\left\{P_{1},P_{2},\cdots ,P_{K}\right\}$.

**Proof:**  Since $A_{c}$ is unambiguous and m>N, one has $\text{rank}\;(A_{c})=N$. It is easy to identify that $\text{rank}\;\left(R_{d}\right)=\text{rank}\;\left(\sum_{i=1}^{M-2P+2}G\left(i\right)G^{H}\left(i\right)\right)$. Referring to the Lemma 1 in [16], we know that given $H\in C^{I\times J}$, the diagonal matrix **Q** has no identical entries on the diagonal, and $\text{rank}\;\left(H\right)=r<I$, then $\text{rank}\;\left(\left[\begin{array}{c} H,QH \end{array}\right]\right)=r+1$. Substituting back into our problem, we find that the diagonal entries of **Φ** are not the same as each other, and $\text{rank}\;\left(b(i)\right)=1$, and thus $\text{rank}\;\left(\left[b(i),\Phi b(i)\right]\right)=2$. If Lemma 1 is applying successively to the submatrices in G(i), one has a) if 2q≥N, then $\text{rank}\;\left(G(i)\right)=N$; b) $\text{rank}\;\left(G(i)\right)=2q$, otherwise. If the latter holds, according to the assumption that the *K* coherent groups are uncorrelated to each other, ${\tilde{R}}_{x}$ has *K* linearly independent rows. Therefore, $\text{rank}\;\left(R_{d}\right)\;=\;\text{rank}\;\left(\sum_{i=1}^{M-2P+2}G\left(i\right)G^{H}\left(i\right)\right)=\text{min}\left\{2qK,N\right\}$. As $2q\geq P_{max}$, one has $2qK\geq KP_{max}\geq N$, and $\text{rank}\;(R_{d})=N$ eventually.              ■

**Remark 1.**  *The parameter m plays a significant role in rank restoration since it determines the array’s DOF and effective aperture. We consider two extreme cases to discuss the choices of m. If* $m\;=\;M\;-\;2P+3-\lceil \frac{P_{max}}{2}\rceil$, *i.e., m achieves its upper bound, then one barely restores the rank deficiency with the minimum number of columns of* B(i), *but this may result in some signals being undetected, especially at low SNRs or for few snapshots. On the other hand, If* m=N+1, *i.e., m achieves its lower bound, then one restores the rank deficiency with redundant columns of* B(i), *but this may cause biased estimates due to the noise subspace being one-dimensional. Based on our simulation results, the proposed method performs well when m lies between these two bounds, away from the extremes. However, the optimum choice of m is still an open problem and is beyond the scope of this paper*.

When m>N and $2q\geq P_{K}$, the non-singularity of the smoothed covariance matrix $\bar{R}$ can be recovered, then the MUSIC algorithm can be employed to estimate the DOAs of coherent signals. More specifically, one can perform the eigen-decomposition of $\bar{R}$ as

(16) $$\begin{array}{cc} \bar{R} & =U_{s}\Sigma_{s}U_{s}^{H}+U_{n}\Sigma_{n}U_{n}^{H} \end{array}$$

where $\Sigma_{s}$ is a diagonal matrix consisting of the *N* largest eigenvalues, and $\Sigma_{n}$ is a diagonal matrix consisting of the m−N smallest eigenvalues. The columns of $U_{s}$ are the eigenvectors corresponding to the *N* largest eigenvalues, while the columns of $U_{n}$ are the eigenvectors corresponding to the m−N smallest eigenvalues. According to the subspace principle, the columns of $U_{s}$ span the signal subspace, which coincides with the range space of **A**, and the signal subspace is orthogonal to the noise subspace spanned by the columns of $U_{n}$. Thus,

(17) $$\begin{array}{c} ∥a^{H}\left(\theta_{i}\right)U_{n}{∥}_{F}^{2}=0,\;i=1,2,\cdots ,N \end{array}$$

This indicates that the DOAs of the coherent signals can be obtained by finding the *N* peaks from the spatial spectrum function

(18) $$\begin{array}{cc} P(\theta ) & =\frac{1}{∥a^{H}\left(\theta \right)U_{n}{∥}_{F}^{2}} \end{array}$$

So far, we have described the proposed algorithm for coherent signal DOA estimation in the presence of unknown mutual coupling. The major steps of the proposed algorithm are summarised as follows:

Step 1. Obtain *L* snapshots of the received signal x(t) at $t=t_{1},t_{2},\cdots ,t_{L}$, and form the following matrix as:

(19) $$\begin{array}{cc} x & =\left[x\left(t_{1}\right),x\left(t_{2}\right),\cdots ,x\left(t_{L}\right)\right] \end{array}$$

Step 2. Calculate the covariance matrix using the above data matrix by

(20) $$\begin{array}{cc} R_{x} & =\frac{1}{L}xx^{H} \end{array}$$

Step 3. Select the middle array and calculate corresponding covariance matrix ${\tilde{R}}_{x}$ according to Equation (6);

Step 4. Utilise rows of ${\tilde{R}}_{x}$ to construct the matrix B(i),i=1,2,⋯,M−2P+2, according to Equation (10);

Step 5. Construct the overall rank-restored matrix $\bar{R}$ as Equation (14);

Step 6. Perform the eigen-decomposition of $\bar{R}$, and obtain the noise subspace $U_{n}$;

Step 7. Scan the direction over [−90°, 90°] with a step size of 0.1°. Calculate the spacial spectrum using Equation (18), and obtain the DOA estimates $\theta_{1},\theta_{2},\cdots ,\theta_{N}$.

Although the mutual coupling information for a sensor array is a cumbersome calculation using electromagnetic simulators and measurement in practice, for some circumstances, such as high precision applications in satellite navigation and airborne early warning radar, it has to be obtained and compensated for a-priori. In the presence of known mutual coupling, we should first eliminate the effects of mutual coupling before applying the matrix reconstruction for rank restoration. To be specific, the following steps have to be carried out:

Step 1. Perform eigen-decomposition of $R_{x}$, one has

(21) $$\begin{array}{cc} R_{x} & ={\tilde{U}}_{s}{\tilde{\Sigma }}_{s}{\tilde{U}}_{s}^{H}+{\tilde{U}}_{n}{\tilde{\Sigma }}_{n}{\tilde{U}}_{n}^{H} \end{array}$$

Since ${\tilde{U}}_{s}$ and CAΓ span the same signal subspace, there holds ${\tilde{U}}_{s}=CA\Gamma T$ where T is a nonsingular matrix.

Step 2. Reconstruct a covariance matrix by compensating for mutual coupling effects as

(22) $$\begin{array}{cc} R_{o} & =C^{-1}{\tilde{U}}_{s}{\tilde{\Sigma }}_{s}{\tilde{U}}_{s}^{H}{\left(C^{H}\right)}^{-1} \end{array}$$

Then, following the similar Steps 4–7 in the unknown mutual coupling case one can resolve the coherent signals.

**Remark 2.**  *Once the mutual coupling information is known, one can compensate the coupling effects by Equation (22), and then the additional information of the auxiliary sensors, which had to be abandoned in the case of unknown mutual coupling, can now be exploited. Compared with Equation (14) using* ${\left\{B(i)\right\}}_{i=1}^{M-2P+2}$, *there are more matrices* ${\left\{B(i)\right\}}_{i=1}^{M}$ *available after mutual coupling compensation, which means that more DOF and a larger effective aperture can be achieved, and improved estimation performance can thus be expected. In fact, our method can handle up to* $M-\lceil \frac{P_{max}}{2}\rceil$ *coherent signals in the presence of known mutual coupling. The identifiability of DOA estimation for the proposed and comparative methods with unknown mutual coupling will be discussed in Section 3.3*.

### 3.3. Separable Signal Number

The coherent signals are resolved with an increased effective array aperture compared with previous techniques. This makes better use of the DOF of the original ULA and allows more signals to be resolved. This motivates us to discuss the number of separable signals of the proposed method. Compared with the standard spatial smoothing (SS) technique, which estimates the coherent signals by averaging subarrays, under unknown mutual coupling [14], the maximum number of signals estimated by our method can be relaxed. If m>N and $2q\geq P_{max}$, the proposed method can estimate a maximum number of $M-2P+2-\lceil \frac{P_{max}}{2}\rceil$ coherent signals since $M-2P+2\geq N+\lceil \frac{P_{max}}{2}\rceil$, while the standard spatial smoothing can estimate at most $M-2P+2-P_{max}$ coherent signals since $P_{max}$ subarrays are required and $M-2P+3-P_{max}>N$. As we can see, the key factor influencing the number of resolvable coherent signals is the maximum number of signals in one coherent group, in other words, the larger $P_{max}$ is, the better our approach performs. Our approach also has better DOA estimation accuracy than SS as will be demonstrated by the simulation results in the next section. The method by Mao *et al.* [15], referred to as matrix reconstruction in data (MRD), can also achieve better DOA estimation accuracy than SS, provided that the effective array aperture is the same, but this comes at a cost of the number of signals which can be resolved. It can resolve at most $\left\lfloor \frac{M-2P+1}{2}\right\rfloor$ coherent signals and the array aperture is fixed regardless of the number of coherent sources. The spatial smoothing techniques are more flexible in this aspect as the number of subarrays and effective array aperture can be varied. Both the algorithms in [14,15] also ignore the conjugate of the received data in rank restoration, which significantly restricts the number of resolvable signals in practice.

Table 1 lists the minimum number of array elements required to resolve a given number of signals by the three methods. For simplicity, we assume that each group has the same number of coherent signals. We can see that our method can use less array elements than the other two algorithms, to estimate the same number of signals.

**Table 1:** Minimum number of array elements required.

Coupling Length	Coherent Signals		Total Signals	Number of Array Elements		
	Groups	Signals in Each Group		SS	MRD	Proposed Method
2	1	2	2	6	7	5
2	2	2	4	8	11	7
3	1	4	4	12	13	10
3	2	3	6	13	17	12
4	1	6	6	18	19	15
4	2	4	8	18	23	16
4	2	6	12	24	31	21
3	3	6	18	28	41	23

## 4. Simulation Results and Discussion

In this section, a series of numerical experiments under different conditions are conducted to examine the performance of the proposed method. Simulations are carried out for a 14-element ULA with half-wavelength spacing between adjacent elements. For simplicity, we assume that all coherent signals have identical power $\sigma_{s}^{2}$, and the input SNR is defined as $10\log_{10}\left(\sigma_{s}^{2}/\sigma_{n}^{2}\right)$. SS, MRD, and the proposed method with known mutual coupling dealt with in Equation (22) are chosen for comparison. The mutual coupling is assumed can be negligible at a distance larger than 1.5λ. Hence, P=3 and the mutual coupling coefficients are assumed to be $c_{0}=1,c_{1}=-0.1545+0.4755j,c_{2}=0.1618-0.1176j$. The accuracy of the DOA estimate is measured from 1000 Monte Carlo runs in terms of the root mean square error (RMSE) which is defined as

(23) $$\begin{array}{cc} \mathrm{RMSE} & =\sqrt{\frac{1}{1000N}\sum_{n=1}^{1000}\sum_{i=1}^{N}{\left({\hat{\theta }}_{i}^{\left(n\right)}-\theta_{i}\right)}^{2}} \end{array}$$

where ${\hat{\theta }}_{i}^{\left(n\right)}$ is the estimate of $\theta_{i}$ for the *n*-th trial, and *N* is the number of coherent signals. Additionally, to assess the overall reliability of all the algorithms, the probability of resolution is defined as

(24) $$\text{Probability}\;\text{of}\;\text{resolution}=\frac{F_{r}}{F}$$

where *F* is the number of trials, and $F_{r}$ is the number of successful estimations for which the absolute DOA estimation errors are within 1°.

### 4.1. DOA Estimation of Coherent Signals From One Group

**Figure 3:**
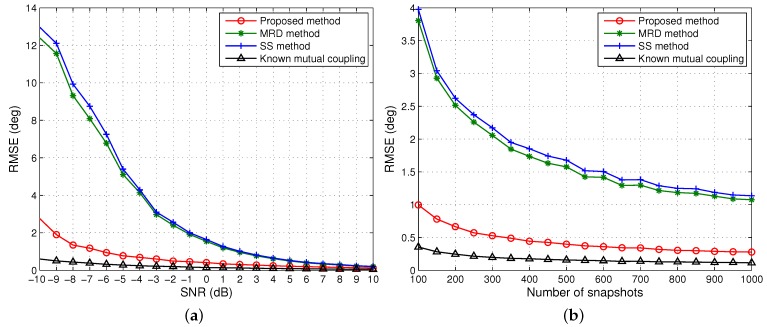
Root mean square error (RMSE) of the direction of arrival (DOA) estimates of one group coherent signals *versus* (**a**) SNR when the number of snapshots is 500; (**b**) the number of snapshots when SNR = 0 dB. Effective array aperture *m* = 5.

Figure 3 depicts the RMSE of DOA estimates of a group of four coherent signals from [−26°, −8°, 9°, 30°]. The fading amplitudes and phases of the coherent signals are [1, 0.8, 0.7, 0.4] and [25.7°, 71.17°, 300.83°, 128.37°], respectively. Here, for fair comparison it is first assumed that the three algorithms have an identical aperture after rank restoration, *i.e.*, *m* = 5. From this figure, we can see that as the SNR and the number of snapshots increase, the RMSE of DOA estimation decreases gradually for all of the methods. The proposed method is significantly superior to MRD and SS, especially at low SNRs and few snapshots, since more column vectors have been utilised for rank restoration with the same array aperture, and is only inferior to the one when mutual coupling is known. Although MRD slightly outperforms SS, both have poor DOA estimation accuracy at low SNRs and few snapshots, and there is a clear discrepancy between these two and the proposed method even for snapshot sizes up to 1000 and beyond at 0 dB as shown in Figure 3b.

**Figure 4:**
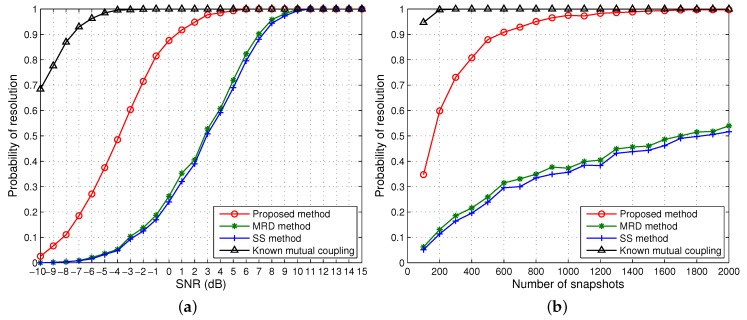
Probability of resolution for one group of coherent signals *versus* (**a**) SNR when the number of snapshots is 500; (**b**) the number of snapshots when SNR = 0 dB. Effective array aperture *m* = 5.

Figure 4 depicts the probability of resolution of successful estimation *versus* input SNR and the number of snapshots for the same scenario as in Figure 3. It can be observed that all methods attain a 100% successful estimation above 11 dB. As the SNR decreases, the probability of successful estimation starts dropping for each method at a certain point, known as SNR threshold, until it eventually becomes zero. With unknown mutual coupling, the proposed method has the lowest SNR threshold followed by MRD and then SS which has the highest SNR threshold. The performance of the proposed method when the mutual coupling is known is also shown for comparison. Evidently the proposed method is superior to the counterparts. When the SNR is fixed at 0 dB, our approach can achieve 100% probability with unknown mutual coupling above 1800 snapshots. Even if the number of snapshots is greater than 2000, the MRD and SS algorithms can barely reach 50% success probability. Consistent with the illustration in Figure 3, MRD has a slightly better performance than SS in terms of probability of resolution *versus* SNR and the number of snapshots.

The simulations above demonstrate the performance of all algorithms with an identical aperture *m* = 5. As discussed in Section 3.3, the effective array aperture after rank restoration by MRD is always halved, in other words, the effective array aperture is fixed and cannot adapt to changes in the number of signals. In contrast, both the proposed and SS algorithm are adaptive to complex situations, such as multiple coherent groups, allowing a trade off between array aperture and rank restoration. To evaluate this advantage, we set *m* = 6 for the proposed and SS algorithm and compare them with the fixed array aperture MRD algorithm, *i.e.*, *m* = 5, while keeping the other configurations the same as the first scenario.

**Figure 5:**
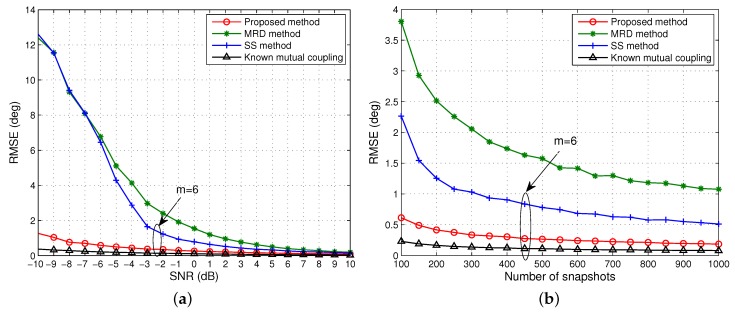
RMSE of the DOA estimates of one group coherent signals *versus* (**a**) SNR when the number of snapshots is 500; (**b**) the number of snapshots when SNR = 0 dB. Effective array aperture *m* = 6 for the proposed and SS method.

**Figure 6:**
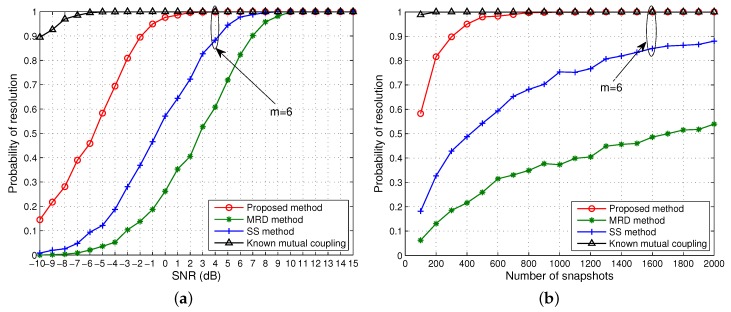
Probability of resolution for one group of coherent signals *versus* (**a**) SNR when the number of snapshots is 500; (**b**) the number of snapshots when SNR = 0 dB. Effective array aperture *m* = 6 for the proposed and SS method.

Figure 5 and Figure 6 show that compared with Figure 3 and Figure 4 the performance of both our approach and SS meliorates, and our approach is still superior to SS and MRD and approaches to the case with known mutual coupling, while the SS method has the largest improvement in the accuracy and reliability. This is mainly because the effective array aperture is improved while keeping the rank completely restored. This group of simulations fully demonstrates that the proposed and SS algorithm have a clear advantage over the MRD algorithm, and the fixed aperture imposed by MRD is not optimal for rank restoration.

### 4.2. DOA Estimation of Coherent Signals From Multiple Groups

We consider two groups of five coherent signals from [−36°, −22°] and [−10°, 5°, 19°] impinge on the ULA. The fading amplitudes of the coherent signals are [1, 0.9] and [1, 0.8, 0.6], while the fading phases are [48.74°, 121.15°] and [189.35°, 35.66°, 283.56°], respectively. The total number of signals is equal to half the elements in the middle array, and thus MRD fails to work. To enable the proposed algorithm and SS work, we select *m* = 6, 7 for rank restoration.

**Figure 7:**
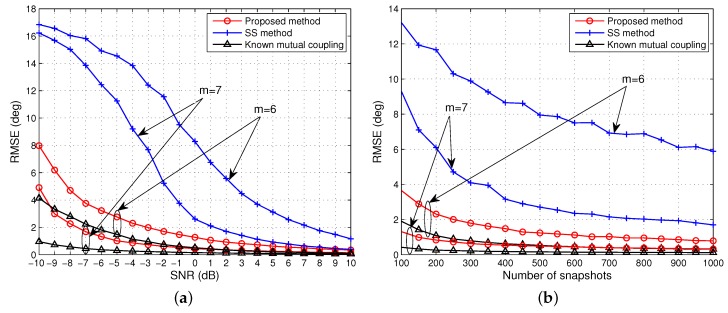
RMSE of the DOA estimates of two groups coherent signals *versus* (**a**) SNR when the number of snapshots is 500; (**b**) the number of snapshots when SNR = 0 dB. Effective array aperture *m* = 6, 7.

Based on the simulation settings, the results of the RMSE *versus* SNR and the number of snapshots are depicted in Figure 7. As shown in this figure, the proposed approach performs the best over the whole range of SNR and the number of snapshots values for coherent signal estimation. When *m* = 6, the proposed algorithm with known or unknown mutual coupling is inferior to the first scenario. One possible explanation is that the fading coefficients in the two scenarios do not have same level of impact for rank restoration in general. SS has larger estimation errors than our method as it only exploits of 4 subarrays for rank restoration while our method effectively utilises twice as many. When *m* = 7, both the proposed and SS algorithm achieve a better performance since the limitation of effective array aperture is relaxed. Although the SS algorithm improves significantly for *m* = 7, it is still notably worse than our proposed method.

**Figure 8:**
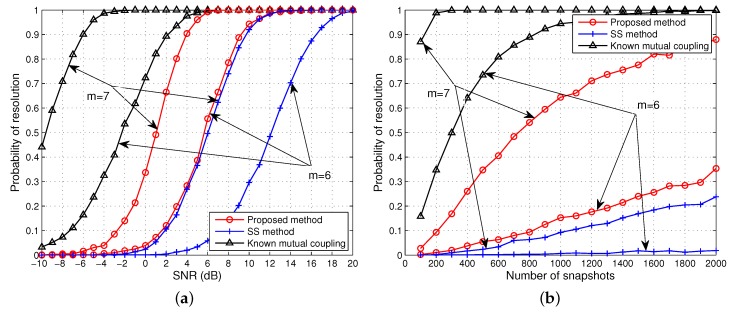
Probability of resolution for two groups of coherent signals *versus* (**a**) SNR when the number of snapshots is 500; (**b**) the number of snapshots when SNR = 0 dB. Effective array aperture *m* = 6, 7.

Next, in Figure 8 we plot the probability of resolution of the two methods by varying the SNR and total number of snapshots. It can be seen that the proposed approach still outperforms the SS algorithms for both configuration *m* = 6, 7. The performance of the SS method improves considerably when *m* = 7 but is strictly worse than our method.

## 5. Conclusions

This paper addresses the problem of DOA estimation when unknown mutual coupling and multipath coexist. An efficient approach has been presented that first circumvents the unknown mutual coupling effect using the output of the selected middle array, and then reconstructs the covariance matrix of the middle array for rank restoration. The proposed method has a better DOA estimation accuracy than the existing methods and can resolve more coherent signals. This is achieved due to two main factors. Firstly replacing standard spatial smoothing in the rank restoration step by a new matrix reconstruction algorithm takes advantage of the information in the off the main diagonal entries of the spatial covariance matrix. Secondly the new matrix reconstruction algorithm for restoring the rank of the coherent signal subspace exploits the conjugate information of the entries of the covariance matrix. These are ignored by standard spatial smoothing techniques and can effectively improve the array aperture or equivalently improve the rank restoration. Extensive simulation results demonstrate the validity and efficiency of the proposed method.

## References

[1] Schmidt R.O. (1986). Multiple emitter location and signal parameter estimation. IEEE Trans. Antenn. Propag..

[2] Roy R., Kailath T. (1989). ESPRIT—Estimation of signal parameters via rotational invariance techniques. IEEE Trans. Acoust. Speech Signal Process..

[3] Friedlander B., Weiss A.J. (1991). Direction finding in the presence of mutual coupling. IEEE Trans. Antenn. Propag..

[4] Pillai S.U., Kwon B.H. (1989). Forward/backward spatial smoothing techniques for coherent signal identification. IEEE Trans. Acoust. Speech Signal Process..

[5] Pasala K.M., Friel E.M. (1994). Mutual coupling effects and their reduction in wideband direction of arrival estimation. IEEE Trans. Aerosp. Electron. Syst..

[6] Adve R.S., Sarkar T.K. (1994). Compensation for the effects of mutual coupling on direct data domain adaptive algorithms. IEEE Trans. Antenn. Propag..

[7] Dandekar K.R., Ling H., Xu G. (2002). Experimental study of mutual coupling compensation in smart antenna applications. IEEE Trans. Wireless Commun..

[8] Sellone F., Serra A. (2007). A novel online mutual coupling compensation algorithm for uniform and linear arrays. IEEE Trans. Antenn. Propag..

[9] Ye Z., Liu C. (2008). On the resiliency of MUSIC direction finding against antenna sensor coupling. IEEE Trans. Antenn. Propag..

[10] Ye Z., Dai J., Xu X., Wu X. (2009). DOA estimation for uniform linear array with mutual coupling. IEEE Trans. Aerosp. Electron. Syst..

[11] Dai J., Xu W., Zhao D. (2012). Real-valued DOA estimation for uniform linear array with unknown mutual coupling. Signal Process..

[12] Bao Q., Ko C.C., Zhi W. (2005). DOA estimation under unknown mutual coupling and multipath. IEEE Trans. Aerosp. Electron. Syst..

[13] Xu X., Ye Z.F., Zhang Y.F. (2009). DOA estimation for mixed signals in the presence of mutual coupling. IEEE Trans. Signal Process..

[14] Dai J., Ye Z. (2011). Spatial smoothing for direction of arrival estimation of coherent signals in the presence of unknown mutual coupling. IET Signal Process..

[15] Mao W., Li G., Xie X., Yu Q. (2014). DOA estimation of coherent signals based on direct data domain under unknown mutual coupling. IEEE Antenn. Wireless Propag. Lett..

[16] Di A. (1985). Multiple source location-a matrix decomposition approach. IEEE Trans. Acoust. Speech Signal Process..

